# The use of Raman spectroscopy to identify and grade prostatic adenocarcinoma *in vitro*

**DOI:** 10.1038/sj.bjc.6601059

**Published:** 2003-07-01

**Authors:** P Crow, N Stone, C A Kendall, J S Uff, J A M Farmer, H Barr, M P J Wright

**Affiliations:** 1 Cranfield Post-Graduate Medical School, Pullman Court, Gloucestershire Royal Hospital, Great Western Road, Gloucester, GL1 3NN, UK; 2 Bristol Royal Infirmary, Marlborough Street, Bristol, BS2 8HW, UK

**Keywords:** prostate cancer, benign prostatic hypertrophy, Raman spectroscopy, Gleason score

## Abstract

Raman spectroscopy is an optical technique, which provides a measure of the molecular composition of tissue. Raman spectra were recorded *in vitro* from both benign and malignant prostate biopsies, and used to construct a diagnostic algorithm. The algorithm was able to correctly identify each pathological group studied with an overall accuracy of 89%. The technique shows promise as a method for objectively grading prostate cancer.

Raman spectroscopy is an optical technique that utilises molecular-specific, inelastic scattering of light photons to interrogate biological tissues (Mahadevan-Jansen *et al*, 1998). When tissue is illuminated with laser light, photons interact with intramolecular bonds present within the tissue. When occurs, the photon donates energy to or receives energy from the bond, producing a change in the bond's vibrational state. When it subsequently exits the tissue, the photon has an altered energy level and, therefore, has a different wavelength compared to the original laser light. This change in the photon's energy is known as the ‘Raman shift’ and is measured in wavenumbers. Photons interacting with different biochemical bonds within the tissue, undergo different Raman shifts, which taken together, form the ‘Raman spectrum’. The Raman spectrum is a plot of intensity against Raman shift, in wavenumbers. As the Raman Shift is inversely proportional to the change in the photons' wavelength, wavenumbers are expressed in units of cm^−1^. The Raman spectrum is a direct function of the molecular composition of the tissue and can therefore give a truly objective picture of the pathology. This is in contrast to Gleason grading, which is subjective and associated with considerable interobserver variation in reporting (Allsbrook *et al*, 2001a, 2001b). A previous study has confirmed that it is possible to record good-quality Raman spectra from prostatic tissue (Crow *et al*, 2002); this study evaluates the ability of Raman spectroscopy to differentiate between different prostatic pathologies *in vitro*.

## MATERIALS AND METHODS

Ethical approval was obtained to take an extra core at prostate biopsy procedures. These biopsies were snap frozen in liquid nitrogen and transferred to a −80°C freezer for storage. A frozen section was taken from each biopsy and processed for standard histological examination, and the remainder of the biopsy was retained at −80°C. The sections were examined by a Consultant Uro-Pathologist and histological diagnosis was made. Those sections containing prostate adenocarcinoma were then examined by a second Consultant Pathologist. In order to minimise the effects of interobserver variation in Gleason scoring, biopsies were only included in the study where an agreement on the Gleason score (GS) was reached between the two pathologists. The position of the prostate cancer within each section was carefully mapped so that the region could subsequently be accurately targeted on the Raman system. Biopsies were classified as benign prostatic hyperplasia (BPH) or adenocarcinoma, with the adenocarcinoma group further split into three groups depending on Gleason score (GS less than seven, GS equal to seven and GS greater than seven).

The remainder of each prostate biopsy, from which the section had been taken, was thawed in preparation for scanning on an optimised Raman system (Renishaw System 1000). The system employs a diode laser, producing 350 mW of near-infrared light at 832 nm. The laser was accurately targeted onto the tissue sample via an ultralong working distance times 80 microscope objective. This produces a laser spot of approximately 30 square microns, which penetrates the tissue up to a depth of 100 *μ*m. The information held within each Raman spectrum, therefore, relates to a cluster of around 30 cells. Using the histological map provided by the pathologist, the laser spot was targeted precisely onto an area of known pathology within each sample. The laser spot was then moved to random locations within this known area, to allow up to 20 spectra to be recorded from each sample. Each Raman spectrum was recorded on the spectrometer using an acquisition period of 20 s. A total of 450 spectra were measured from biopsies taken from 27 different patients, 14 with BPH and 13 with adenocarcinoma.

The spectral data were loaded onto the Matlab platform (Mathworks Inc., Natick, Massachusetts), which, in conjunction with the PLS Toolbox (eigenvector, Manson, Washington), was used to perform the principal component fed, linear discriminant analysis required to construct a diagnostic algorithm. The accuracy of the algorithm, in correctly predicting the histological diagnosis for each spectrum, was tested using ‘leave one spectrum out’ crossvalidation. This ensured that the data from the spectrum, which was having its diagnosis predicted by the algorithm, had not been used in constructing the algorithm being tested. A qualitative analysis was also undertaken by comparing the mean of the spectra recorded from BPH with the mean of the spectra recorded from all prostate cancers.

## RESULTS

Figure 1

**Figure 1:**
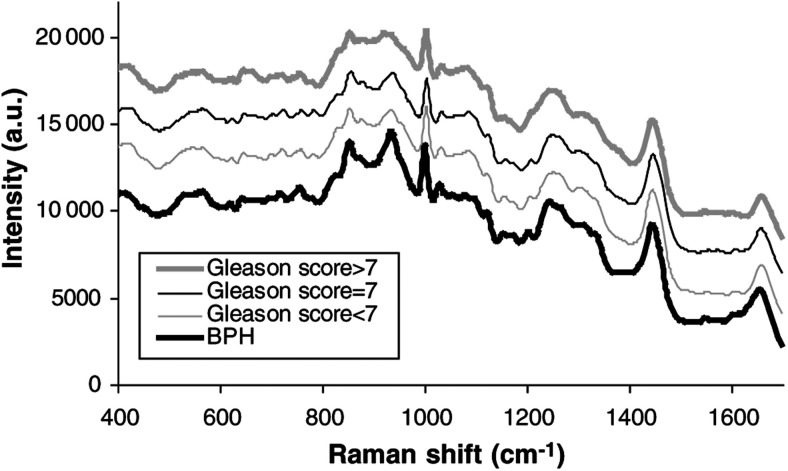
The Mean Raman spectra for each pathological group studied.

shows mean, spectra for each pathological group studied. In the interests of clarity, the spectra have been arranged so that they are equally spaced with respect to the intensity axis. Figure 2

**Figure 2:**
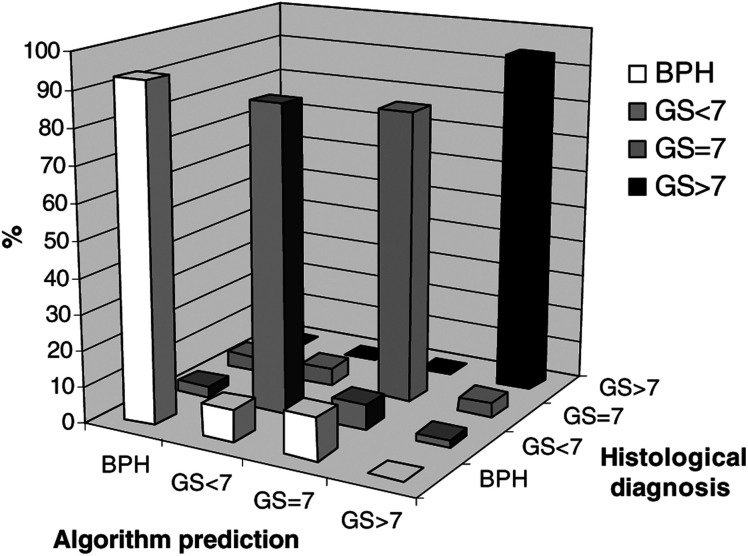
Bar chart and grid demonstrating the prediction power of the diagnostic algorithm.

illustrates the prediction power of the algorithm, with the algorithm-predicted diagnosis plotted against the confirmed histological diagnosis. The large columns along the diagonal row correspond to the correct prediction of histological diagnosis, with the other small columns representing misdiagnoses by the algorithm. The rows of Table 1

**Table 1:** Confirmed histological diagnosis against the diagnosis predicted by the algorithm

		Algorithm			
Prediction		**BPH**	GS<7	GS=7	**GS>7**
Histological diagnosis	BPH	192	6	9	0
	GS <7	11	99	6	0
	GS =7	10	6	70	0
	GS >7	0	1	2	44

show the confirmed histological diagnosis for each spectrum measured. The table columns show the diagnosis predicted by the algorithm for each spectrum measured. By reading across each row, the number of correct and incorrect algorithm predictions, for each histological diagnosis, can be viewed. Table 2

**Table 2:** Sensitivity and specificity achieved by the diagnostic algorithm for each pathological group

	**BPH**	**Gleason score <7**	**Gleason score =7**	**Gleason score >7**
Total no. of spectra/(total no. of patients)	207 (14)	116 (6)	86 (4)	47 (3)
Sensitivity	93%	85%	81%	94%
Specificity	92%	96%	96%	100%

shows how the algorithm's predictive accuracy translates to sensitivity and specificity for each pathological group studied.

Figure 3

**Figure 3:**
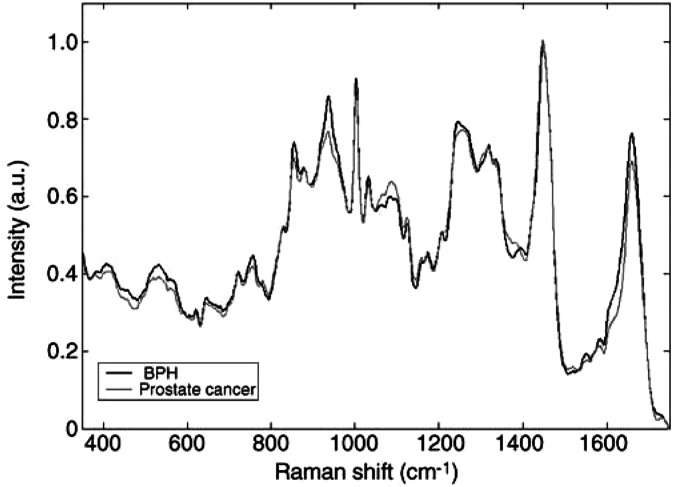
The mean Raman spectra recorded from BPH and all prostate cancers studied.

shows the mean spectra recorded from BPH and all prostate cancers studied. Where visual differences in the peak heights of the two spectra were noted, the relevant literature was consulted for information as to the nature of these peaks (Hartman *et al*, 1973; Manoharan *et al*, 1995; Boustany *et al*, 1996; Mahadevan-Jansen, 1996; Manfait *et al*, 2000; Stone *et al*, 2002). Although the biochemical designations of many peaks remain uncertain, the differences between the two mean spectra suggest that prostate cancer has increased levels of nucleic acids and reduced levels of glycogen, when compared to BPH.

## DISCUSSION

This study shows that Raman spectroscopy can be used to accurately identify BPH and three different grades of prostatic adenocarcinoma *in vitro*. Snap-frozen samples were used in order to reproduce *in vivo* conditions as closely as possible, however, work carried out by our group on other tissues has confirmed that the technique can achieve similar results with formalin-fixed specimens (Stone, 2001). The technique therefore shows promise as a pathology tool providing a rapid, objective method for diagnosing and grading prostate cancer. Work is ongoing to increase the sample size of the study and also to determine whether the technique is able to provide prognostic information for individual patients with prostate cancer. Potential *in vivo* applications include use, via a needle probe, to guide prostatic biopsy and allow intraoperative assessment of tumour resection margins.

The qualitative findings of reduced glycogen content and increased nucleic acid content in malignant, compared to benign pathologies, fit in with the findings of previous studies of the larynx (Stone *et al*, 2000), colon (Feld *et al*, 1995), oesophagus (Bakker Schut *et al*, 1997) and cervix (Mahadevan-Jansen *et al*, 1998). These findings provide an insight into the type of molecular differences, which allow the technique to differentiate between the different pathologies. The use of multivariate spectral analysis ensures that the diagnostic algorithm is constructed using all the molecular information available from the Raman spectra. Retrieving quantitative biochemical information from the Raman spectra of prostatic tissue, however, currently remains a prospect for the future.
